# A Rare Case of Giant Cell Tumour of the Medial Epicondyle of the Humerus Managed With Curettage and Bone Grafting

**DOI:** 10.7759/cureus.43437

**Published:** 2023-08-13

**Authors:** Abhiram Awasthi, Nareshkumar Dhaniwala, Shounak Taywade, Mohit Dadlani, Shivshankar Jadhav

**Affiliations:** 1 Orthopaedic Surgery, Jawaharlal Nehru Medical College, Datta Meghe Institute of Medical Sciences, Wardha, IND

**Keywords:** giant cell curettage, distal humerus gct, medial epicondyle, humerus, giant cell tumour of bone

## Abstract

Giant cell tumours (GCTs) of the medial epicondyle of the humerus are rare. These are generally benign tumours but have the potential to be locally aggressive. They can invade the adjacent joint or the surrounding soft tissues or, in rare cases, cause distant metastasis. Locally aggressive GCTs are generally treated with wide resection, curettage, and bone grafting, followed by joint reconstructions. Here we present a case of a 49-year-old female with a history of swelling over the medial epicondyle of the humerus for six months. The patient was diagnosed with a locally aggressive GCT and was managed with wide excision of the tumour followed by sandwich bone grafting. A two-year follow-up of the patient shows no signs of recurrence. The patient is pain-free and has decent elbow function.

## Introduction

Giant cell tumours (GCTs) are rare bone tumours that involve the epiphyseal-metaphyseal region of the long bones [1,2]. These account for approximately 6% of all primary bone tumours [3] and 20% of all benign bone tumours [4]. It most commonly occurs in the third to fourth decade of life and has a slight female predominance [5,6]. The most common sites for GCTs are the distal end of the femur, proximal tibia, and the distal end of the radius. Still, their occurrence in the medial epicondyle of the distal humerus is rare [7,8].

GCTs of the bone can spread till the epiphysis without eroding the joint, or they can sometimes erode the cortex and spread into the joint or surrounding soft tissue or rapidly grow in size and migrate into the metaphysis. Surgical intervention is the treatment of choice, most frequently with either curettage alone or combined with other chemical or thermal adjuvants. Wide excision and repair have been advised for tumours that have penetrated the cortex (Campanacci Grade 3 lesions) and are close to joint capsules.

Despite surgical excision of the affected tissues and reconstruction, the long-term functional effects of these reconstructions still need to be better understood. Several surgical techniques and adjuvant therapies have been suggested to reduce the likelihood of recurrence in these tumours. The literature reports that the recurrence rate after simple curettage, with or without bone grafting, ranges from 17% to 55% [9,10]. It has been demonstrated that local adjuvant therapy effectively reduces these recurrence rates. According to the literature, recurrence rates in GCTs treated with curettage and local adjuvant therapy range from 6% to 25% [11,12].

## Case presentation

**Figure 1:**
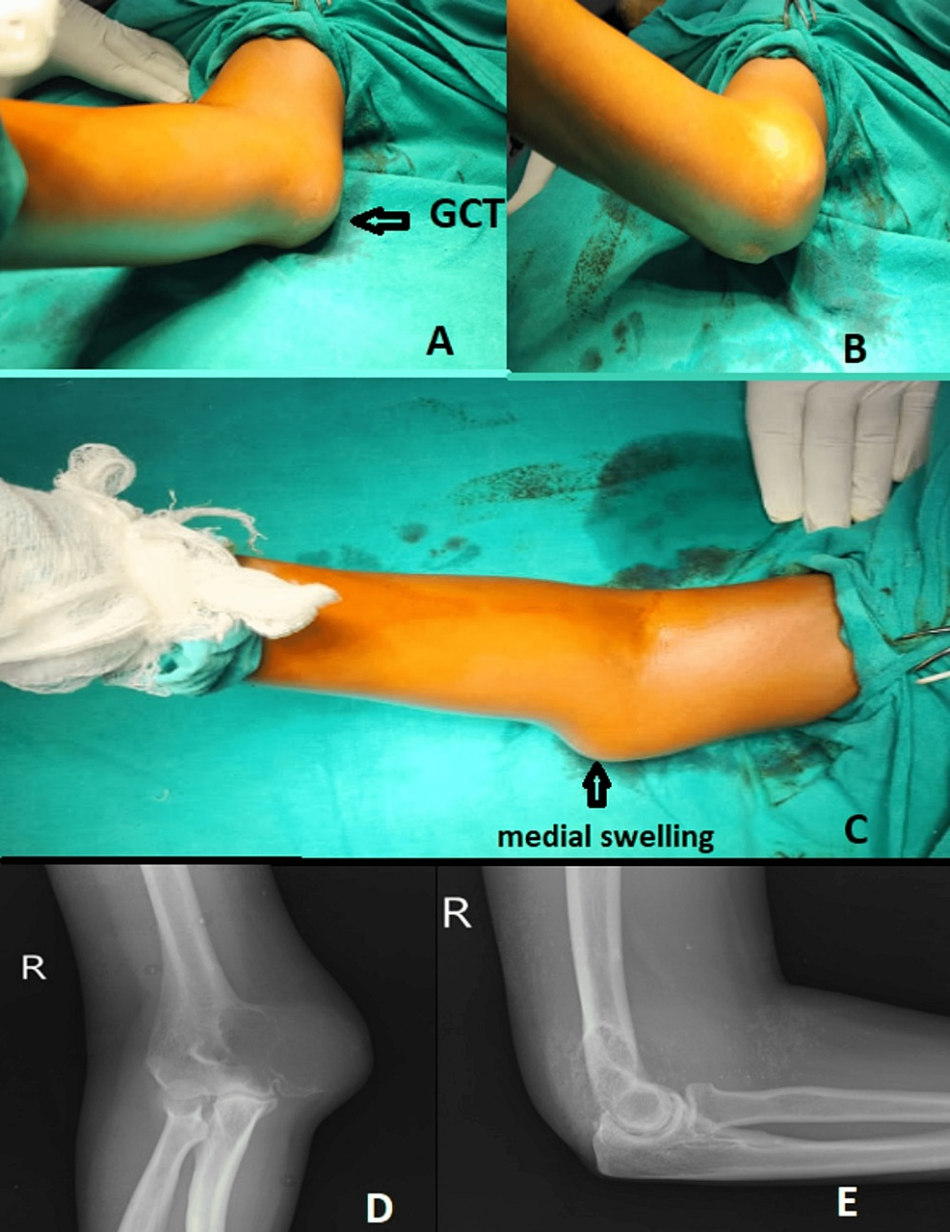
Clinical photographs and X-rays of right elbow in anteroposterior and lateral views showing expansile lesion involving the medial epicondyle of right humerus (A, B, and C) Clinical photographs of the swelling over the right medial aspect of the elbow. (D and E) Anteroposterior and lateral views of the right elbow, showing bony swelling with a soap bubble appearance over the medial epicondyle of the humerus. GCT: giant cell tumour

A 49-year-old female presented to the OPD with pain and swelling over her right elbow for six months. She also complained of decreased range of movements in the past four months. Her symptoms were not associated with trauma. On examination, there was diffuse swelling over the medial aspect of the right elbow, around 8 × 10 cm, with localized tenderness over the bulge. There was a fixed flexion deformity of 30 degrees with the inability to pronate or supinate the forearm. X-rays of the right elbow joint were done, and they showed a lytic expansile lesion over the medial epicondyle of the humerus with a soap bubble appearance (Figure 1).

**Figure 2:**
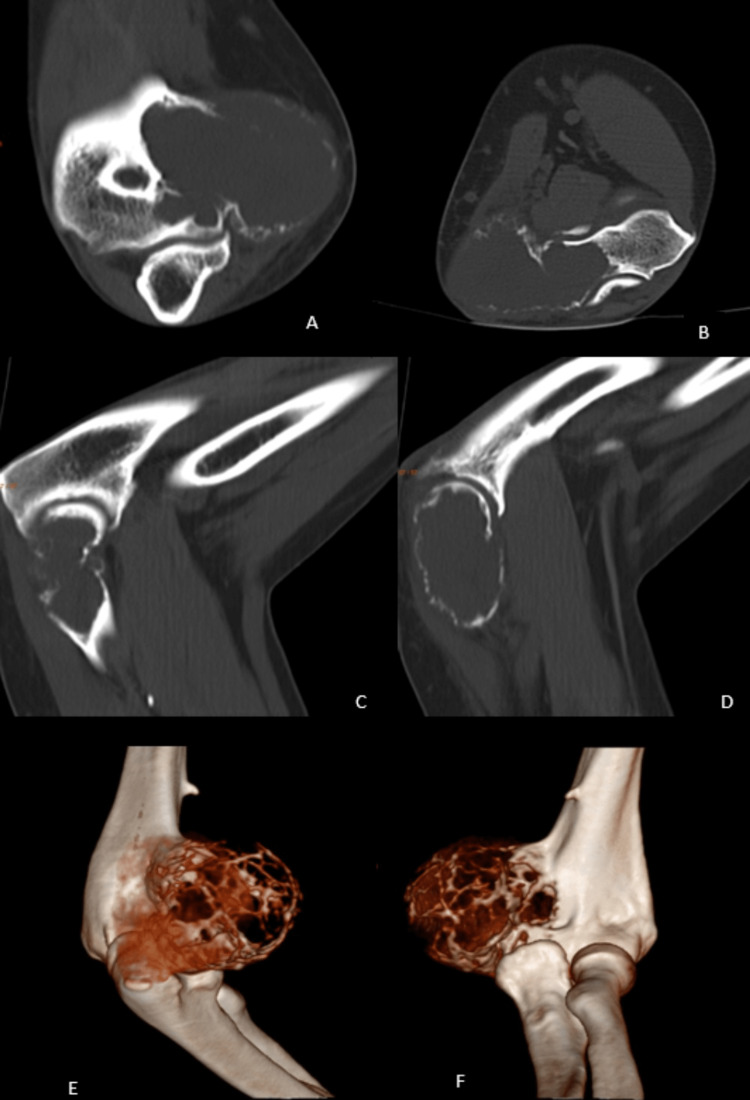
CT scans of the right elbow with 3D reconstruction (A) Coronal section of the right elbow swelling. (B) Axial section of the right elbow swelling. (C and D) Sagittal sections of right elbow swelling. (E and F) 3D reconstructed images of the bony swelling.

CT scans of the right elbow reported a locally destructive lytic lesion involving the medial epicondyle of the humerus with involvement of the medial soft tissue and trochlea measuring 4.6 × 3.5 × 4.5 cm with cortical erosions (Figure 2).

**Figure 3:**
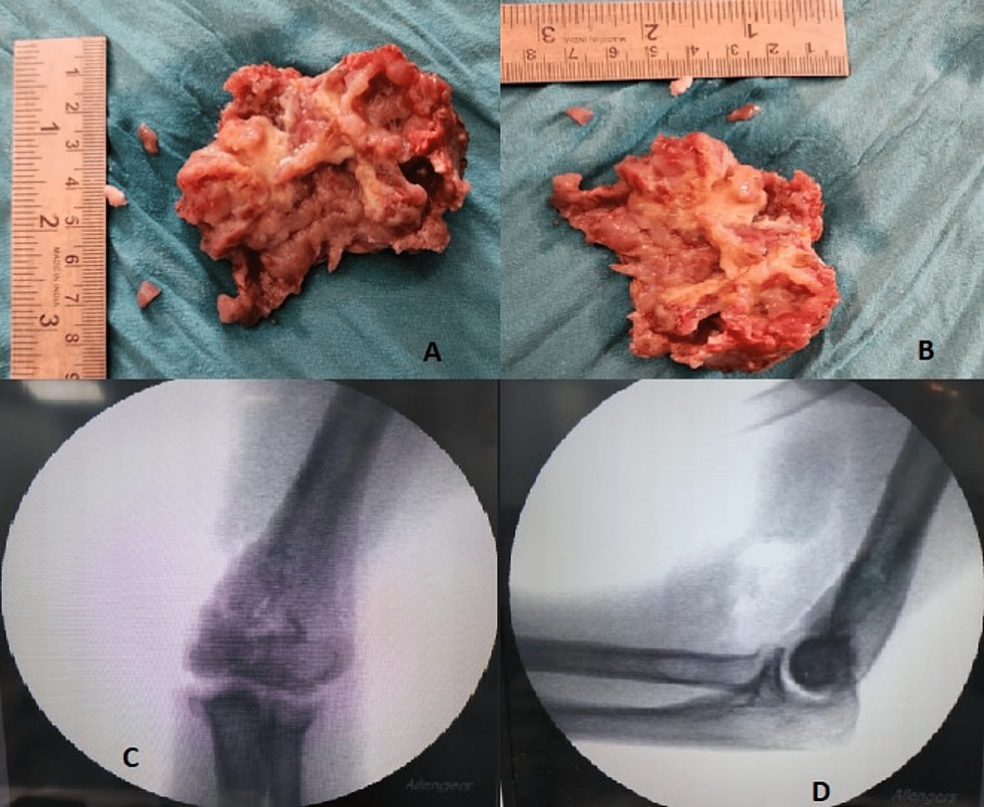
Intraoperative images with C-arm photographs (A and B) Resected tumour mass with measurements. (C and D) anteroposterior and lateral views of the right elbow after curettage and bone grafting

A biopsy was then performed that confirmed the mass to be a GCT, showing multiple isolated and small clustered populations of osteoclastic giant cells with a small fragment of monophasic stromal cells that carry ovoid to spindle benign nuclei. Further CT scans of the thorax and USG abdomen were performed to rule out any malignancies, which were negative. A joint preserving procedure was chosen for the patient considering her age and the joint was not involved. Under supraclavicular and axillary nerve block, A medial approach to the elbow was taken. The ulnar nerve was identified and was found to be intact. A plane was made between the brachialis and the triceps muscle, and directly the tumour edges were exposed. After careful dissection, the tumour was removed en bloc. Curettage of the surrounding healthy bone tissues was done with the help of a high-speed burr machine. Then the cavity's interior surface was subsequently painted with 90% phenol using a sterile applicator to perform chemical cauterization. The cavity was cleansed with pulsed saline lavage after the phenol had been in place for five minutes. The cavity was filled with an autologous cancellous bone graft from the ipsilateral iliac crest and bone cement. The final alignment of the elbow was found to be stable; hence further plating was avoided (Figure 3).

**Figure 4:**
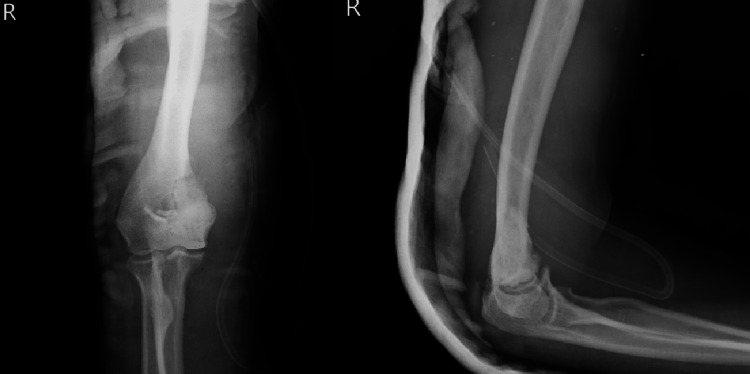
Immediate post-operative X-ray Anteroposterior and lateral views of the right elbow

After the surgical procedure, X-Rays were done, which showed good joint space and no signs of tumour tissue remaining (Figure 4).

**Figure 5:**
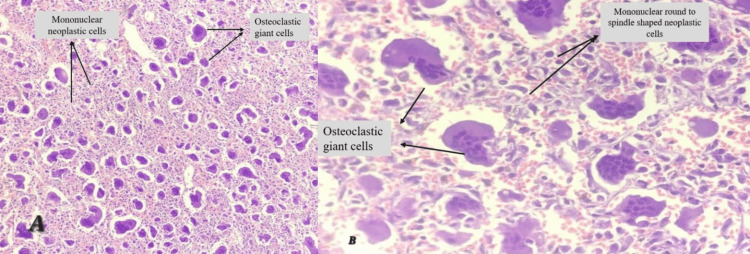
Histopathological slides of the tumour (A) Low-power view of GCT showing many osteoclastic giant cells along with mononuclear neoplastic cells. (B) High-power view of GCT showing osteoclastic giant cells along with mononuclear round, oval to spindle-shaped neoplastic cells.

Histopathological studies showed many multinucleated osteoclastic giant cells along with mononuclear neoplastic cells, which are characteristic of a GCT (Figure 5). 

**Figure 6:**
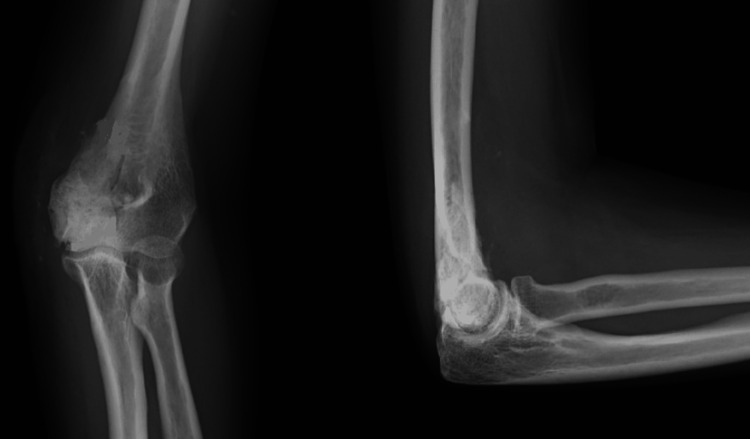
Post-operative X-ray at the end of two years Anteroposterior and lateral views of the right elbow at the end of two years

After surgery, the elbow was protected for six weeks by an above-elbow cast. Six weeks after the surgery, active elbow mobilization was started, and strengthening exercises were initiated. Three months following the procedure, the patient went back to work, and 20 to 100 degrees of flexion was achieved at the end of two months. The patient remained in follow-up for two years, and at the end of the second year, there were no signs of recurrence, and the patient was pain-free with sufficient elbow movements to do her daily activities. X-rays at the end of two years showed good incorporation of bone graft and no signs of recurrence (Figure 6).

## Discussion

A GCT of the bone usually presents as an eccentric expansile lytic lesion on X-rays with a characteristic soap bubble appearance. They show cortical thinning, but a breach in the cortex occurs in later stages. X-rays and computed tomography scans help evaluate the cortex breach and adjacent joint involvement. These tumours have a characteristic histological presentation of mononuclear stromal cells and multinucleated giant cells. The gold standard of care for treating GCTs of the bone is surgical resection and intralesional curettage followed by bone grafting or wide resection with joint arthroplasties. GCTs have been observed to recur typically within the first two years of resection [13]. Depending on whether a bone graft was utilized to fill the defect or not, simple curettage shows recurrence rates ranging from 27% to 55% [13,14]. The only drawback is that the patients frequently need to endure prolonged protected weight bearing in lesions treated with bone grafts until the graft is integrated. Several cytotoxic agents, such as phenol, have been used to chemically cauterize any remaining cancerous cells in the tumour cavity to decrease recurrence rates. As a local adjuvant to wide excision, Phenol has been shown to reduce the recurrence rates of GCT [14]. Bone curettage followed by bone cementing has been found to significantly decrease the recurrence rates of GCTs. In a study conducted by Persson et al. [15] in 1984, it was observed that the recurrence rates decreased from 55% to 14% when polymethyl methacrylate (PMMA) was used instead of acrylic cement. In a recent study by Saiz et al. [11], they reported recurrence rates of only 12.5% in a group of 36 patients who underwent intralesional curettage, high-speed burring, electrocauterization, phenol cauterization, and PMMA application.

Following wide excision of a GCT, the most effective additional treatment approach remains a topic of ongoing discussion. According to Blackley et al. [16], high-speed burr excision played a vital role, with other treatments providing only minor benefits. Turcotte et al. [17], on the other hand, argued that adjuvants could not compensate for poor surgical technique and found no notable distinction between adjuvant methods and filler materials. The efficacy of phenol and electrocautery in preventing recurrence has yielded conflicting results [17-19]. We posit that combining multiple modalities maximizes tumour eradication. Moreover, the use of polymethyl methacrylate (PMMA) promptly reinforces the subchondral bone, promoting patient mobility. The cement polymerization generates temperatures ranging from 47 to 57 degrees Celsius, potentially contributing to the thermal necrosis of any residual tumour cells [20].

Elbow prostheses are another option that can be used after wide excision, but considering the patient's young age, we went for wide excision and bone grafting sandwiched with PMMA. Endoprostheses are also susceptible to infections, and the polyethene liner may wear out, resulting in prosthetic loosening. Elbow prostheses are, therefore, suitable for elderly patients with limited functional demands but less ideal for young and active persons. In young people, joint-preserving treatment should therefore be sought. It has lower risks for complications, is less expensive, and may provide better functional long-term results than wide resection and end prosthetic reconstruction.

## Conclusions

We conclude that in young aged patients, wide excision of the tumour with curettage and thermal cauterization followed by bone grafting and PMMA bone cement augmentation by sandwich technique is a better option than en bloc excision and joint replacement as it provides a more pain-free and mobile joint with fewer chances of infection and recurrence.
